# Nondestructive Detection of Pesticide Residue (Chlorpyrifos) on Bok Choi (*Brassica rapa* subsp. Chinensis) Using a Portable NIR Spectrometer Coupled with a Machine Learning Approach

**DOI:** 10.3390/foods12050955

**Published:** 2023-02-23

**Authors:** Ravipat Lapcharoensuk, Chawisa Fhaykamta, Watcharaporn Anurak, Wasita Chadwut, Agustami Sitorus

**Affiliations:** 1 Department of Agricultural Engineering, School of Engineering, King Mongkut’s Institute of Technology, Ladkrabang, Bangkok 10520, Thailand; 2 Research Center for Appropriate Technology, National Research and Innovation Agency (BRIN), Subang 41213, Indonesia

**Keywords:** NIR spectroscopy, machine learning, bok choi, pesticide

## Abstract

The contamination of agricultural products, such as vegetables, by pesticide residues has received considerable attention worldwide. Pesticide residue on vegetables constitutes a potential risk to human health. In this study, we combined near infrared (NIR) spectroscopy with machine learning algorithms, including partial least-squares discrimination analysis (PLS-DA), support vector machine (SVM), artificial neural network (ANN), and principal component artificial neural network (PC-ANN), to identify pesticide residue (chlorpyrifos) on bok choi. The experimental set comprised 120 bok choi samples obtained from two small greenhouses that were cultivated separately. We performed pesticide and pesticide-free treatments with 60 samples in each group. The vegetables for pesticide treatment were fortified with 2 mL/L of chlorpyrifos 40% EC residue. We connected a commercial portable NIR spectrometer with a wavelength range of 908–1676 nm to a small single-board computer. We analyzed the pesticide residue on bok choi using UV spectrophotometry. The most accurate model correctly classified 100% of the samples used in the calibration set in terms of the content of chlorpyrifos residue on samples using SVM and PC-ANN with raw data spectra. Thus, we tested the model using an unknown dataset of 40 samples to verify the robustness of the model, which produced a satisfactory F1-score (100%). We concluded that the proposed portable NIR spectrometer coupled with machine learning approaches (PLS-DA, SVM, and PC-ANN) is appropriate for the detection of chlorpyrifos residue on bok choi.

## 1. Introduction

Bok choi (*Brassica rapa* subsp. Chinensis) is a horticultural product; both its stems and leaves are consumed, with or without cooking. Pesticides are often used to control insect attacks during cultivation, as well as to maintain productivity. However, the pesticides used to protect plants during cultivation cannot be removed and thus become residuals. Therefore, the detection of pesticide residue on agricultural products is important as a modern food safety issue to prevent such residue from entering the body [1]. In addition, pesticide residue can cause serious environmental pollution and pose risks to human health if consumed. The Ministry of Public Health of Thailand reported that the most commonly detected pesticide residue in bok choi is chlorpyrifos [2] and that bok choi had the highest prevalence of pesticide residues in products on the market. Many researchers have also discovered chlorpyrifos residues in vegetable and fruit samples from local markets and supermarkets in Thailand [3,4,5,6]. According to the National Food Safety Standard (GB 2763-2016), the maximum residue limit (MRL) for chlorpyrifos in vegetables is set to 0.1 mg/kg [7]. The Ministry of Public Health of Thailand published the Notification on Food Containing Pesticide Residues in the Royal Gazette to ban chlorpyrifos residues in food products, stating that the MRL of chlorpyrifos should be zero for all products [8]. Sankorn et al. [1] reported that the Thai Department of Agriculture discovered excessive use of pesticides during agricultural cultivation and tried to reduce their use through various mitigation campaigns. A warning should be placed on the consumption of bok choi due to safety concerns. Although policies have been implemented with support from regulations, infrastructure, such as the technology needed to rapidly detect and simultaneously screen for residue on agricultural products, must be applied.

Generally, pesticide residue is detected on agricultural products through a variety of complex methods and instruments. The methods involved in measuring the concentration of pesticide residue include gas chromatography (GC), which was used to detect pesticide residue in green tea leaves [9]; high-performance liquid chromatography (HPLC), which was used to detect insecticides in cucumber and eggplant [10]; and GC-LC mass spectrometry (GC-LC-MS), which was used for several agricultural products [11]. However, these techniques are destructive, involve complicated sample preparation, and must be performed in an advanced laboratory. As such, these techniques cannot be used for the rapid and real-time screening of agricultural production. Therefore, a detection approach that is nondestructive, simple to prepare, and reliable for detecting pesticide residue on agricultural products needs to be developed to independently monitor products.

Currently, one of the most popular nondestructive measurement technologies used to monitor agricultural products is near infrared (NIR) spectroscopy (NIRS) [12,13]. This method works based on the absorption or reflectance of radiation in the near-infrared region of the electromagnetic spectrum in the range of 780–2500 nm toward organic functional groups, including single bonds of CH, OH, and NH, and the double bond of CO [14]. This method has been successfully applied for qualitative and quantitative analyses, especially to detect pesticide residue in agricultural products, including vegetables and fruits. Several studies on the feasibility of NIRS in detecting pesticide residue on fruit and vegetable products are listed in Table 1.

Although pesticide residue (chlorpyrifos) has been reported in agricultural products in several studies [24,25,26], to the best of our knowledge, the nondestructive detection of chlorpyrifos residue in leafy vegetables that are consumed fresh has not been reported. Only research by Ngo et al. [15], investigated the application of a handheld spectrometer to estimate pesticide residues on leafy vegetables, including lettuce, Oriental mustard, and bok choi. Hence, in this study, we aimed to classify the absence or presence of chlorpyrifos on bok choi (*Brassica rapa* subsp. Chinensis) using a portable NIR spectrometer combined with a machine learning approach.

## 2. Materials and Methods

### 2.1. Sample Preparation

A total of 120 bok choi (*Brassica rapa* subsp. Chinensis) samples were cultivated separately in 2 small greenhouses. In the first greenhouse, we planted 60 samples, which were not sprayed with any pesticide from the beginning of cultivation to the harvest stage. These samples were used as the chlorpyrifos-free group (CF). In the second greenhouse, samples were inoculated with commercial liquid chlorpyrifos 40% EC (C_9_H_11_C_l3_NO_3_PS). The pesticide was diluted with distilled water, for a final chlorpyrifos concentration in the spray of 2 mL/L. Every 7 days during cultivation, we sprayed the plants with dosage rates of approximately 300 mL/m^2^ using a backpack pressure sprayer (OLD-8L-04, FONTE, Bangkok, Thailand). The concentration and rate of spraying were in accordance with the instructions of the pesticide manufacturer, which were specified on the product label. The spraying was stopped 3 days before harvesting. We called this group the chlorpyrifos residue (CR) group. All samples were harvested 45 days after cultivation. Prior to each NIR spectrum collection, samples were left to reach an equilibrium temperature of 25 °C in a laboratory environment.

### 2.2. NIR Spectra Data Collection

**Figure 1:**
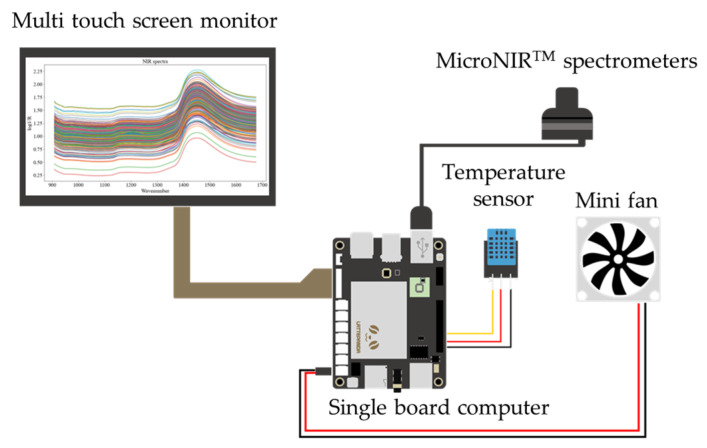
Schematic of portable NIR spectrometer.

In this study, we connected a commercial portable NIR spectrometer (MicroNIR^TM^ spectrometer) (Viavi Solutions Inc., Santa Rosa, CA, USA) to a small single-board computer (DFR0419, LattePanda, Shanghai, China). The dispersing element used by this portable NIR was a linear variable filter (LVF). A multitouch screen monitor was used as the assembly of both the input (touch panel) and output (display) device. The internal heat of the portable meter was ventilated with a small fan that was controlled. Figure 1 shows a schematic of the portable NIR spectrometer. Each sample was placed between the window of the MicroNIR^TM^ spectrometer and the aluminum plate lid. The reason for using an aluminum plate lid behind each sample was to return the signal to the spectrometer, specifically to improve the signal-to-noise ratio [27]. The NIR spectra of the bok choi samples were acquired in a wavelength range of 908–1676 nm in diffuse reflection mode with an interval of 6.2 nm. Data acquisition was performed using MicroNIR^TM^ Pro v2.2 software (Viavi Solutions Inc., Santa Rosa, CA, USA). NIR spectral data were collected from 3 positions: the head, middle, and tail on the leaves of each vegetable sample. The scanning was performed in triplicate at each position, and we averaged the results. Therefore, we obtained data on the NIR spectrum from both the CF and CR groups, totaling 360 (i.e., 180 spectra of CF and 180 spectra of CR) for training the models.

### 2.3. Determination of the Real Value of Pesticide Residue

For the determination of pesticide residue in the bok choi, all samples were analyzed for chlorpyrifos using the UV spectrophotometric method, following Harshit et al. [28], with the required modifications. The purified chlorpyrifos (98.5% purity and 10 mg) was weighed with an electrical balance and then transferred to a 100 mL volumetric flask. We poured ethyl acetate (99.8% purity) into a 100 mL volumetric flask to create a 0.1 mg/mL solvent for the stock solution of chlorpyrifos. Working standard solutions of different concentrations were prepared (i.e., 0.2–3.0 μg/mL) by diluting the stock solution with ethyl acetate. A UV spectrophotometer (GENESYS 10S UV-VIS, Thermo Fisher Scientific, Waltham, MA, USA) and quartz cuvettes were used for absorbance measurements at 277 nm. Each experiment was performed in three replicates. A calibration curve was created by plotting the absorbance versus the concentration of the working standard solutions.

To extract the chlorpyrifos from the vegetables, we finely cut and chopped the leaves of each vegetable from the NIR spectrum collection. Twenty grams of bok choi and 50 mL of ethyl acetate were transferred to a conical flask and then blended by shaking. Sodium bicarbonate (5 g) was placed in a conical flask, and the mixture was shaken for 5 min. After that, we added magnesium sulfate (15 g) and shook the mixture on a mechanical flask shaker for 1 h. The mixture was filtered with Whatman paper (No. 40), and the filtrate was centrifuged at 1500 rpm for 5 min. The sample was evaporated at 80 °C up to 2 mL with a rotary evaporator. Ethyl acetate and cyclohexane were mixed with a ratio of 1:1 to obtain 20 mL of mixture. Finally, the solution was poured into a 10 mL volumetric flask, and ethyl acetate was used for dilution up to the mark. The absorbance value of the sample was measured with 5 replications following the same procedure described in the section above. Chlorpyrifos residue was determined using a calibration curve and a regression equation of the linearity graph.

### 2.4. Machine Learning Process

#### 2.4.1. Data Preprocessing

Normally, NIR spectra are influenced by weather, environment, humidity, temperature, instrument, and human factors [29,30]. Many types of external interference and noise might be reflected in the NIR spectra, which consist of 125 waveband points. Therefore, the NIR spectra were preprocessed to solve these issues and improve the performance of the predictive model before modeling [31,32,33]. NIR spectral data were preprocessed via 7 techniques: Savitzky–Golay smoothing (SGS), mean normalization (MN), standard normal variate and detrending (SNV&D), baseline collection (BC), multiplicative scatter correction (MSC), and Savitzky–Golay first (D1) and second (D2) derivatives [30,34,35]. We used the un-preprocessed spectra (RS) and each preprocessed spectrum via the above techniques to train the calibration models.

Principal component analysis (PCA) is a classical unsupervised learning algorithm used for dimensionality reduction [36]. The original spectrum is transformed into a smaller number of uncorrelated variables or principal components (PCs). In our study, PCA was performed to reduce the dimensionality of the NIR spectral data, and new variables (i.e., the first 20 PCs) were applied to the input layer of the neural network for the hybrid principal component–artificial neural network (PC-ANN). Software for multivariate analysis (Unscrambler X Version 10.5.1, Camo, Norway) was used for spectral preprocessing and the PCA procedure.

#### 2.4.2. Modeling and Evaluation of Model Performance

After data preprocessing, we applied four machine learning (ML) algorithms for classification: partial least-squares discrimination analysis (PLS-DA), support vector machine (SVM), artificial neural network (ANN), and PC-ANN to develop the calibration models. Modeling was performed using the Python programming language with the Scikit-learn (Version 1.0.2) packages [37]. For PC-ANN, the first 20 principal component scores (PCs) were used as the input layer of the neural network instead of the original NIR spectra. The optimal number of PCs was determined with a 5-fold cross-validation of PC-ANN in which the error of classification did not increase after adding one more PCs. Using PCs as the input nodes for the ANN reduced both training time and redundancy in the original NIR spectra. In recent years, PC-ANN has been successful in modeling NIR spectroscopy [38,39,40,41,42]. The samples were split into 288 for training (80%) and 72 for testing (20%). The hyperparameters of each ML method were defined to train the calibration models. We found the optimal hyperparameters by performing 5-fold cross-validation experiments on the training dataset. The effective models were selected when the appropriate model provided the best maximized accuracy for the classification of CF and CR. Optimization of the hyperparameters was performed using the GridSearchCV command of the Scikit-learn module [37]. Table 2 presents the predefined parameters for performing the GridSearchCV of PLS-DA, SVM, ANN, and PC-ANN. The performance of the classification models was evaluated by assessing the accuracy, precision, recall, and F1-score, which we calculated using Equations (1)–(4).

(1) $$\mathrm{accuracy}=\frac{\mathrm{TP}+\mathrm{TN}}{\mathrm{TP}+\mathrm{TN}+\mathrm{FP}+\mathrm{FN}}$$

(2) $$\mathrm{precision}=\frac{\mathrm{TP}}{\mathrm{TP}+\mathrm{FP}}$$

(3) $$\mathrm{recall}=\frac{\mathrm{TP}}{\mathrm{TP}+\mathrm{FN}}$$

(4) $$F1-\mathrm{score}=2\;\times \frac{\mathrm{precision}\times \mathrm{recall}}{\mathrm{precision}+\mathrm{recall}}$$

Here, TP and TN represent the numbers of true positives and negatives, respectively; FP and FN are the numbers of false positives and negatives, respectively. In machine learning, accuracy, precision, recall, and F1-score are the common metrics used to evaluate the performance of classification models, especially for binary problems [43,44]. These parameters have long been used in the evaluation of scientific models and engineering applications [45] and in the evaluation of the performance of NIR spectroscopy combined with machine learning [46,47,48,49]. Accuracy is the ratio between the correctly classified samples and the total number of samples in the evaluated dataset [50]. Precision is the probability of the correct detection of positive values, and recall indicates the ability to discriminate between classes [44]. The F1-score is the harmonic mean of precision and recall; thus, the F1-score maintains a balance between precision and recall for classifiers [44].

#### 2.4.3. Validation of Model with Unknown Sample

To evaluate the performance of the model in the real world and in an overfitting test, we used the calibrated models to classify unknown samples of bok choi. A total of 40 unknown samples were purchased from local markets in Bangkok province (Thailand), and the NIR spectra of these samples were collected using the portable NIR spectrometer. Then, the pesticide residue levels on the vegetables were analyzed using the UV spectrophotometric method. The candidate models from the four algorithms were applied to predict the pesticide residue on the unknown samples, and the prediction performance was evaluated in terms of accuracy, precision, recall, and F1-score.

## 3. Results and Discussion

### 3.1. Spectra of Samples

**Figure 2:**
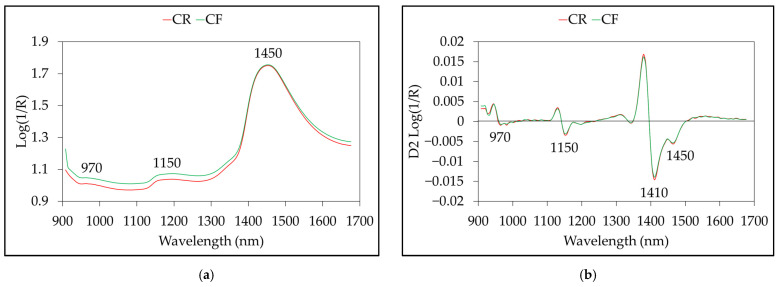
Average NIR spectra fromchlorpyrifos-free (CF) and chlorpyrifos residue (CR) groups: (**a**) raw spectra; (**b**) Savitzky–Golay second derivative spectra.

The average NIR spectra from the CF and CR groups are shown in Figure 2a. The spectra of both sample groups had a similar shape but differed in absorbance intensity across the spectral region. A total of 125 waveband points were acquired in one wavelength from 908 to 1676 nm. The wavebands at 970 and 1450 nm are the second and first overtones of O-H stretching of water, respectively; the absorption observed at 1152 nm is the C-H stretching of the second overtone of CH_3_ [51,52]. Figure 2b shows the spectra processed by the Savitzky–Golay second derivative, with a five-point window and second-order polynomial. New absorbance peaks were revealed at 1410 nm in the second-derivative spectra, which were hidden in the raw spectra. The apparent peak at 1410 nm corresponded to the combination of the C-H stretching of methylene [53,54].

### 3.2. Results of Real Chlorpyrifos Residue Value

The statistical results for chlorpyrifos residue on samples from analysis with the UV spectrometric method are shown in Table 3. For the calibration stages, chlorpyrifos residues were not detected on the CF group samples. For the CR group, the concentration of chlorpyrifos was between 0.011 and 2.184 mg/kg, and the mean was 1.120 ± 0.532 mg/kg. We applied the developed model to 40 unknown samples from local markets and supermarkets and detected the absence (CF group) and presence (CR group) of chlorpyrifos residues on 15 and 25 samples, respectively. For the CR group, the concentration of chlorpyrifos was between 0.022 and 1.596 mg/kg, and the mean was 1.25 ± 0.37 mg/kg. The chlorpyrifos residues on some of the samples from the local markets and supermarkets were above the MRL of the National Food Safety Standard (GB 2763-2016) (0.1 mg/kg) [7].

### 3.3. Principal Componant Analysis

**Figure 3:**
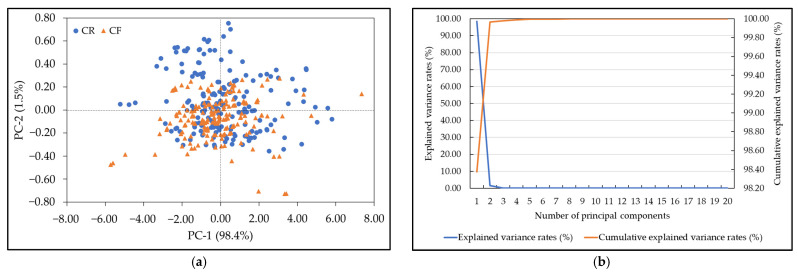
PCA for the chlorpyrifos-free (CF) and chlorpyrifos residue (CR) groups. (**a**) Score plot of PC1 against PC2; (**b**) explained variance rates and cumulative explained variance rates of the first 20 PCs.

We employed PCA to extract the hidden information inside the NIR spectrum and reduce the dimensionality of the spectral data from 125 to 20. The first 20 PCs accounted for 99.99% of the total variance in the NIR spectra. Figure 3 shows the PCA results for the CF and CR groups. Figure 3a shows plots of the first two PCs, where PC-1 and PC-2 explain 98.4% and 1.5% of the total variance in the NIR spectra, respectively. The distribution of the CF group significantly overlapped that of the CR group, which created difficulties in distinguishing the CR and CF groups with PCA. This phenomenon has occurred in many previous studies, although high-performance NIR models with machine learning algorithms have been developed to address this issue [55,56]. Therefore, more PCs were necessary for training the PC-ANN [55]. Figure 3b displays a line plot of the explained variance rates and the cumulative explained variance rates of the first 20 PCs. The explained variance of the 20 PCs was approximately 0.00004%, and the cumulative explained variance rate was 99.99931%. This indicated that these 20 PCs covered all the NIR spectral information, and the PC-ANN model could be developed with the first 20 PCs.

### 3.4. Classification of Vegetables with Machine Learning

Table 4 shows the results of the CF and CR classification from the calibration stage of the samples using several machine learning algorithms. The F1-score of the classification of the presence or absence of pesticides in the samples using the PLS-DA algorithm was between 0.94 and 0.99. The most accurate identification using the PLS-DA algorithm with various preprocessing methods achieved an F1-score of 0.99 in the training and testing stages. We obtained a similar value by applying raw data and baseline correction preprocessing with the PLS-DA algorithm. The results of this study are in line with those of Jamshidi, Mohajerani, and Jamshidi [21], who reported that the PLS-DA algorithm performed well in measuring and detecting diazinon residues in cucumbers using Vis/NIR in the range of 450–1000 nm. Employing the SVM algorithm, the best F1-score was obtained when using raw spectral data rather than preprocessing (1.0 at the training and testing stages). The SVM algorithm also correctly predicted three classes of chlorpyrifos residue contents on filter paper (<100, 100–300, and >300 mg/kg) with 89.29% accuracy [57]. The ANN algorithm with the full wavelength (125 nm) produced the best F1-score by preprocessing the first derivative: 0.83 for the training stage and 0.92 for the testing stage. Finally, the application of the ANN algorithm that used 20 PC inputs produced the best F1-score (100%) without preprocessing in the training and testing stages. The optimal hyperparameters used for machine learning are presented in Table 5.

The results showed that the most accurate machine learning algorithms were SVM and PC-ANN using raw spectral data to identify pesticide residues on bok choi. The classification of the presence or absence of pesticides on samples using the SVM and PC-ANN algorithms achieved 100% accuracy, precision, and recall in the training and testing stages. Thus, the integration of a portable NIR spectrometer with a machine learning approach (SVM or PC-ANN) could be effectively used to classify the absence or presence of pesticide residues on bok choi. In this study, the SVM algorithms and the PC-ANN classifier could accurately discriminate pesticide residue down to a minimum concentration of 0.01 mg/L.

### 3.5. X-Loading and Regression Coefficient

**Figure 4:**
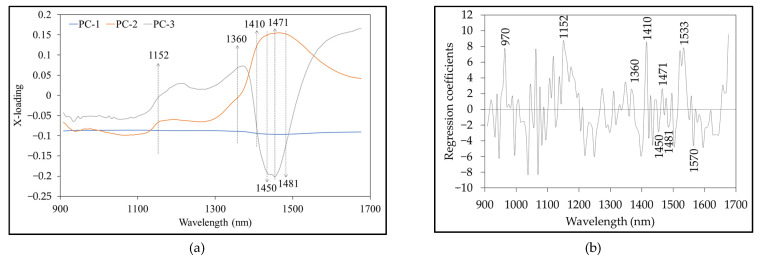
X-loading (**a**) and regression coefficient (**b**) plots.

Figure 4 shows the X-loading plot of the first three PCs from PCA and the regression coefficient plot from the best PLS-DA model. The peaks and valleys with high absolute values of the X-loading weights and regression coefficients represent the vibration of the band at a particular wavelength that influenced the classification of the presence or absence of pesticides in bok choi. High X-loading peaks were obtained at 1152, 1360, 1410, 1450, 1471, and 1481 nm (Figure 4a). Table 6 shows the corresponding absorption bands from the X-loading and regression coefficients. These peaks also occurred in the regression coefficient plot (Figure 4b). We observed high regression coefficient peaks at 970, 1152, 1360, 1410, 1450, 1471, 1481, 1540, and 1570 nm, which corresponded to the vibration bands of H_2_O (970 and 1450 nm), CH_3_ (1152, 1360, and 1410 nm), CONHR (1471 nm), CONH_2_ (1481 nm), C=H (1533 nm), and -CONH- (1570 nm) [51]. The wavelength of 1410 nm correlates with the combination of a single bond of the CH stretching of methylene [53,54]. In addition, 1360 and 1471 nm are associated with methyl and NH primary amides, respectively. According to Rodriguez et al. (2020), these wavelengths (1360, 1410, 1450, 1471, 1540, and 1570 nm) contribute to the detection of chlorpyrifos-methyl [54]. In addition, Sánchez et al. (2010) suggested that absorption in the 1360 and 1480 nm wavelength regions correlates with CH and NH absorption, both of which can indicate the presence or absence of pesticide residues of organophosphates, organochlorides, carbamates, pyrethroids, pyrimidine compounds, dicarboximides, thiazoles, and natural residues on peppers [23].

### 3.6. Validation of Model with Unknown Samples

Table 7 presents the results of the validation of the model on unknown samples. The PLS-DA, SVM, and PC-ANN models performed better than the ANN model in detecting the presence or absence of pesticides in bok choi. The PLS-DA, SVM, and PC-ANN algorithms showed satisfactory performance, with an accuracy rate of 100%. In addition, the F1-score, representing the average harmonic of precision and recall for the three models (PLS-DA, SVM, and PC-ANN), was superior (100%). The qualitative results of the independent validation testing obtained in this study showed the ability of this method to differentiate samples with different levels of pesticide residue, from 0.02 to 1.44 mg/L. Accordingly, these results prove that this model has robustness that has been scientifically shown to be directly applicable at the industrial level because of the satisfactory results.

In contrast, the performance of the ANN algorithm generated from the full spectrum was inferior. When using an independent dataset, it could only detect the presence or absence of pesticide residue in 38% of the sample. The performance of this ANN model in distinguishing positive samples was poor, but its sensitivity was high (100%), so the F1-score was 50%. This showed that the ANN model with a full spectrum was unable to satisfactorily distinguish false positives and false negatives in the sample data. This may have been due to overfitting in the model calibration stage, which caused inconsistent model performance when testing on an independent dataset. Janik et al. [58] reported a similar finding when using full-spectrum Vis/NIR to predict the total anthocyanin concentration in red grape homogenates. The ANN algorithm that uses the full spectrum tends to experience overfitting because it uses excessive input scores as inputs.

## 4. Conclusions

We investigated the detection of a residual pesticide (chlorpyrifos) on bok choi (*Brassica rapa* subsp. Chinensis) using a portable NIR spectrometer with a machine learning approach. The results showed that the combination of NIR spectroscopy and machine learning is useful for effectively classifying the absence or presence of pesticide residues. All machine learning algorithms (i.e., PLS-DA, SVM, ANN, and PC-ANN) achieved an accuracy of between 0.92 and 1.00 in the calibration stage. For the in-field operation of the model, we evaluated the performance of the calibration models of the PLS-DA, SVM, and PC-ANN algorithms on an unknown dataset using accuracy, precision, recall, and F1-scores, with each reaching 100%. Finally, we recommend using the PLS-DA, SVM, and PC-ANN classification algorithms with the full spectrum and raw data to detect the presence or absence of chlorpyrifos residues on bok choi before consumption.

## Figures and Tables

**Table 1:** Summary of studies employing Vis and NIR spectroscopy to detect pesticide residue on horticultural products.

Agro-Products (Source)	Residue	Wavelength (nm)	Pesticide Residue Range (mg/kg)
Lettuce, Oriental mustard, Bok choy [15]	Indoxacarb, chlorantraniliprole, emamectin benzoate	340–840	<0.01–0.56
Chinese kale, cabbage, green chili spur pepper [1]	Profenofos	800–2500	0.53–106.28
Cabbage [7]	Chlorpyrifos, carbendazim	350–2500	0.1–100
Tomato [16,17]	Profenofos	350–1100	0.0–42.9
Melon [18]	Chlorothalonil, imidacloprid, pyraclostrobin	348–1141	1.0
Lettuce leaves [19]	Fenvalerate, chlorpyrifos	950–1650	1.0–10
Cucumber [20,21]	Diazinon	450–1000	0.0–32
Oranges [22]	Dichlorvos	350–1800	1.0–1.25
Peppers [23]	Mixed pesticides	400–1700	0.01–1.05

**Table 2:** Predefined parameters for performing GridSearchCV command on 5-fold cross validation.

Model	Hyperparameter	Tuning Range
PLS-DA	n_components	1–20
SVM	kernel degree gamma	linear, poly, rbf, sigmoid 2–7 0.001–0.09
ANN	activation hidden layer sizes learning rate learning rate initial	identity, logistic, tanh, relu 100, 110, 120, (100, 100), (110, 110), (120, 120), (100, 110, 100), (110, 120, 110) constant, invscaling, adaptive 0.001, 0.01, 0.1
PC-ANN (PCs = 20)	activation hidden layer sizes learning rate learning rate initial	identity, logistic, tanh, relu 10, 11, 12, (10, 10), (11, 11), (12, 12), (10, 11, 10), (11, 12, 11) constant, invscaling, adaptive 0.001, 0.01, 0.1

**Table 3:** Concentration of chlorpyrifos residues on samples for calibration and prediction stages.

Sample	Sample Group	Max (mg/kg)	Min (mg/kg)	Mean (mg/kg)	SD (mg/kg)
Calibration	Chlorpyrifos-free (CF) (*n* = 60)	n.d. *	n.d.	n.d.	n.d.
	Chlorpyrifos residues (CR) (*n* = 60)	2.184	0.011	1.120	0.532
Unknown	Chlorpyrifos-free (CF) (*n* = 15)	n.d.	n.d.	n.d.	n.d.
	Chlorpyrifos residues (CR) (*n* = 25)	1.596	0.022	1.385	0.410

* Not detected.

**Table 4:** Comparison of results among classification models at the calibration stage.

Model	Preprocessing	Training				Testing			
		Accuracy	Precision	Recall	F1-Score	Accuracy	Precision	Recall	F1-Score
PLS-DA	RS	0.99	0.98	1.00	0.99	0.99	1.00	0.97	0.99
	SGS	0.97	0.97	0.97	0.97	0.97	1.00	0.95	0.97
	MN	1.00	0.99	1.00	1.00	0.97	1.00	0.95	0.97
	SNV&D	0.99	0.99	1.00	0.99	0.97	1.00	0.95	0.97
	BC	0.99	0.98	1.00	0.99	0.99	1.00	0.97	0.99
	MSC	0.99	0.99	1.00	0.99	0.97	1.00	0.95	0.97
	D1	0.99	0.99	0.99	0.99	0.96	0.97	0.95	0.96
	D2	0.99	0.99	0.99	0.99	0.93	0.90	0.97	0.94
SVM	RS	1.00	1.00	1.00	1.00	1.00	1.00	1.00	1.00
	SGS	0.99	1.00	0.99	0.99	0.99	1.00	0.97	0.99
	MN	1.00	1.00	1.00	1.00	0.97	0.97	0.97	0.97
	SNV&D	1.00	1.00	1.00	1.00	0.97	0.97	0.97	0.97
	BC	1.00	1.00	1.00	1.00	0.97	1.00	1.00	1.00
	MSC	1.00	1.00	1.00	1.00	0.97	0.97	0.97	0.97
	D1	0.51	0.00	0.00	0.00	0.47	0.00	0.00	0.00
	D2	0.51	0.00	0.00	0.00	0.47	0.00	0.00	0.00
ANN	RS	0.73	0.88	0.52	0.65	0.81	1.00	0.63	0.77
	SGS	0.61	0.63	0.50	0.56	0.61	0.71	0.45	0.55
	MN	0.54	1.00	0.07	0.13	0.50	1.00	0.05	0.10
	SNV&D	0.67	1.00	0.33	0.50	0.64	1.00	0.32	0.48
	BC	0.61	0.60	0.65	0.62	0.71	0.73	0.71	0.72
	MSC	0.69	1.00	0.30	0.46	0.66	1.00	0.24	0.46
	D1	0.84	0.90	0.77	0.83	0.92	0.97	0.87	0.92
	D2	0.87	0.81	0.96	0.88	0.88	0.85	0.96	0.88
PC-ANN (PCs = 20)	RS	1.00	1.00	1.00	1.00	1.00	1.00	1.00	1.00
	SGS	0.98	0.98	0.97	0.98	0.96	0.97	0.97	0.96
	MN	0.99	0.99	0.99	0.99	0.97	0.94	1.00	0.97
	SNV&D	0.97	0.95	0.99	0.97	0.94	0.89	1.00	0.94
	BC	1.00	1.00	1.00	1.00	0.97	0.97	0.97	0.97
	MSC	0.99	0.99	0.99	0.99	0.97	0.94	1.00	0.97
	D1	0.51	0.51	1.00	0.67	0.47	0.47	1.00	0.64
	D2	0.49	0.00	0.00	0.00	0.53	0.00	0.00	0.00

RS: raw spectral, SGS: Savitzky–Golay smoothing, MN: mean normalized, SNV&D: standard normal variate + 1st derivative, BC: baseline correction, MSC: multiplicative scattering correction, D1: 1st derivative, D2: 2nd derivative, PLS-DA: partial least-squares discriminant analysis, SVM: support vector machine, ANN: artificial neural network, PC: principal component.

**Table 5:** Optimal hyperparameters used for machine learning.

Model	Preprocessing	Hyperparameter
PLS-DA	RS	n_components = 11
SVM	RS	kernel = poly, degree = 6, gamma = 1
ANN	D1	activation = identity, hidden layer sizes = 100, learning rate = invscaling, learning rate initial = 0.001
PC-ANN (PCs = 20)	RS	activation = relu, hidden layer sizes = (11, 11), learning rate = adaptive, learning rate initial = 0.1

**Table 6:** Corresponding absorption bands from X-loading and regression coefficients.

Wavelength (nm)	Bond Vibration/Functional Group (Structure)	Reference
970	O-H str. second overtone (H_2_O)	[51,52]
1152	C-H str. second overtone (CH_3_)	[51,52]
1360	2 × C-H str. + C-H def. (CH_3_)	[51]
1410	2 × C-H str. + C-H def. (CH_3_)	[51,53,54]
1450	O-H str. first overtone (H_2_O)	[51,52]
1471	N-H str. first overtone (CONHR)	[51]
1481	N-H str. first overtone (CONH_2_)	[51]
1533	C-H str. first overtone (C=H)	[51]
1570	N-H str. first overtone (-CONH-)	[51]

**Table 7:** Results of model validation on unknown sample.

Model	Processing Spectra	Independent Validation			
		Accuracy	Precision	Recall	F1-Score
PLS-DA	RS	1.0	1.0	1.0	1.0
SVM	RS	1.0	1.0	1.0	1.0
ANN	D1	0.38	0.38	1.0	0.5
PC-ANN (PCs = 20)	RS	1.0	1.0	1.0	1.0

## Data Availability

The data are available from the corresponding author.
